# Bis[(1*RS*,2*RS*)-4,4′-(1-aza­niumyl-2-hy­droxy­ethane-1,2-di­yl)dipyridinium] tris­[tetra­chloridopalladate(II)]

**DOI:** 10.1107/S1600536812050817

**Published:** 2012-12-22

**Authors:** Jose J. Campos-Gaxiola, Alberto Baez-Castro, Adriana Cruz-Enriquez, Herbert Hopfl, Miguel Parra-Hake

**Affiliations:** a Facultad de Ingenieria Mochis, Universidad Autonoma de Sinaloa, Fuente Poseidon y Prol. A. Flores S/N, CP 81223, C.U. Los Mochis, Sinaloa, Mexico; b Centro de Investigaciones Quimicas, Universidad Autonoma del Estado de Morelos, Av. Universidad 1001, CP 62210, Cuernavaca, Morelos, Mexico; c Centro de Graduados del Instituto Tecnologico de Tijuana, Blvd. Industrial S/N, Col. Otay, CP 22500, Tijuana, B.C., Mexico

## Abstract

The asymmetric unit of the title compound, (C_12_H_16_N_3_O)_2_[PdCl_4_]_3_, consists of a 4,4′-(1-aza­niumyl-2-hy­droxy­ethane-1,2-di­yl)dipyridinium dication and one and a half tetra­chloridopalladate(II) anions; the latter has inversion symmetry. In the cation, the pyridinium rings attached to the central 1-aza­niumyl-2-hy­droxy­ethane fragment show an *anti* conformation, as indicated by the central C—C—C—C torsion angle of −178.1 (4)°, and they are inclined to one another by 25.7 (2)°. In the crystal, the cations and anions are linked through N—H⋯Cl and O—H⋯Cl hydrogen bonds. There are also π–π contacts [centroid–centroid distance = 3.788 (3) Å] and a number of C—H⋯O and C—H⋯Cl inter­actions are present, consolidating the formation of a three-dimensional structure.

## Related literature

For potential applications of organic–inorganic hybrid materials with magnetic, optical and electrical properties, see: Yao *et al.* (2010 ▶); Sanchez *et al.* (2011 ▶); Pardo *et al.* (2011 ▶). For related tetra­chloridopalladate(II) compounds, see: Kumar *et al.* (2006 ▶); Adams *et al.* (2005 ▶, 2006 ▶); Maris (2008 ▶). For the synthesis of the ligand, see: Campos-Gaxiola *et al.* (2012 ▶).
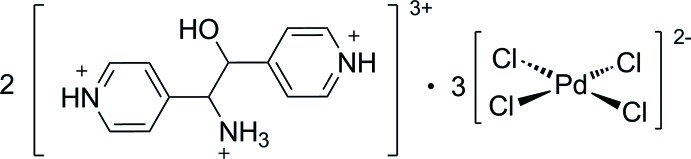


## Experimental

### 

#### Crystal data

(C_12_H_16_N_3_O)_2_[PdCl_4_]_3_
*M* *_r_* = 1181.16
Triclinic, 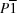

*a* = 7.6970 (7) Å
*b* = 7.7339 (7) Å
*c* = 15.7254 (13) Å
α = 84.541 (2)°
β = 81.314 (2)°
γ = 78.717 (1)°
*V* = 905.40 (14) Å^3^
*Z* = 1
Mo *K*α radiation
μ = 2.40 mm^−1^
*T* = 100 K
0.29 × 0.22 × 0.17 mm

#### Data collection

Bruker SMART CCD area-detector diffractometer
Absorption correction: multi-scan (*SADABS*; Sheldrick, 1996 ▶) *T*_min_ = 0.543, *T*_max_ = 0.686
5043 measured reflections
3143 independent reflections
2927 reflections with *I* > 2σ(*I*)
*R*_int_ = 0.019

#### Refinement

*R*[*F* ^2^ > 2σ(*F* ^2^)] = 0.032
*wR*(*F* ^2^) = 0.077
*S* = 1.07
3143 reflections
232 parameters
6 restraints
H atoms treated by a mixture of independent and constrained refinement
Δρ_max_ = 1.23 e Å^−3^
Δρ_min_ = −0.48 e Å^−3^

### 

Data collection: *SMART* (Bruker, 2000 ▶); cell refinement: *SAINT-Plus* (Bruker, 2001 ▶); data reduction: *SAINT-Plus*; program(s) used to solve structure: *SHELXS97* (Sheldrick, 2008 ▶); program(s) used to refine structure: *SHELXL97* (Sheldrick, 2008 ▶); molecular graphics: *ORTEP-3* (Farrugia, 2012 ▶) and *Mercury* (Macrae *et al.*, 2008 ▶); software used to prepare material for publication: *publCIF* (Westrip, 2010 ▶).

## Supplementary Material

Crystal structure: contains datablock(s) I, global. DOI: 10.1107/S1600536812050817/su2540sup1.cif

Structure factors: contains datablock(s) I. DOI: 10.1107/S1600536812050817/su2540Isup2.hkl

Additional supplementary materials:  crystallographic information; 3D view; checkCIF report

## Figures and Tables

**Table 1:** Hydrogen-bond geometry (Å, °)

*D*—H⋯*A*	*D*—H	H⋯*A*	*D*⋯*A*	*D*—H⋯*A*
O1—H1′⋯Cl5	0.84 (4)	2.22 (4)	3.047 (3)	168 (4)
N1—H1*A*⋯Cl3^i^	0.86 (3)	2.62 (2)	3.355 (4)	145 (4)
N1—H1*A*⋯Cl4^i^	0.86 (3)	2.54 (4)	3.203 (4)	135 (4)
N1—H1*B*⋯Cl6^ii^	0.86 (4)	2.49 (5)	3.310 (4)	160 (4)
N1—H1*C*⋯Cl1^ii^	0.86 (2)	2.22 (2)	3.080 (3)	177 (6)
N2—H2′⋯Cl2^iii^	0.84 (4)	2.35 (4)	3.137 (4)	157 (4)
N3—H3′⋯Cl5^iv^	0.84 (4)	2.44 (4)	3.150 (4)	143 (4)
N3—H3′⋯Cl6^iv^	0.84 (4)	2.71 (5)	3.353 (4)	135 (3)
C4—H4⋯Cl5^v^	0.95	2.64	3.406 (5)	139
C6—H6⋯O1^vi^	0.95	2.54	3.454 (6)	161
C9—H9⋯Cl3^ii^	0.95	2.78	3.599 (5)	145
C10—H10⋯Cl2^vii^	0.95	2.75	3.649 (5)	159
C11—H11⋯Cl1	0.95	2.61	3.486 (5)	154
C11—H11⋯Cl1^viii^	0.95	2.80	3.422 (5)	124

Symmetry codes: (i) 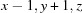; (ii) 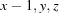; (iii) 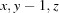; (iv) 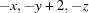; (v) 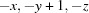; (vi) 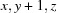; (vii) 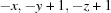; (viii) 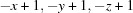.

**Fig. 1.**
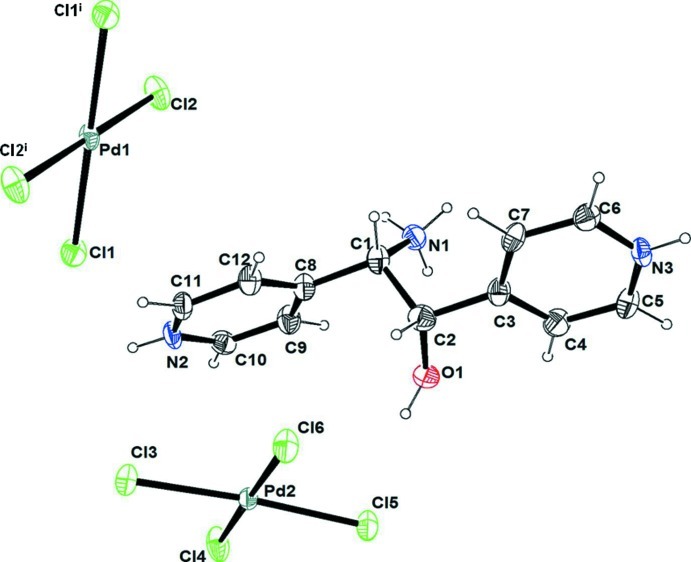
The molecular structure of the asymmetric unit of the title compound, with the atom-labelling. Displacement ellipsoids are drawn at the 50% probability level. [symmetry code: (i) -x + 1, -y + 2, -z + 1].

**Fig. 2.**
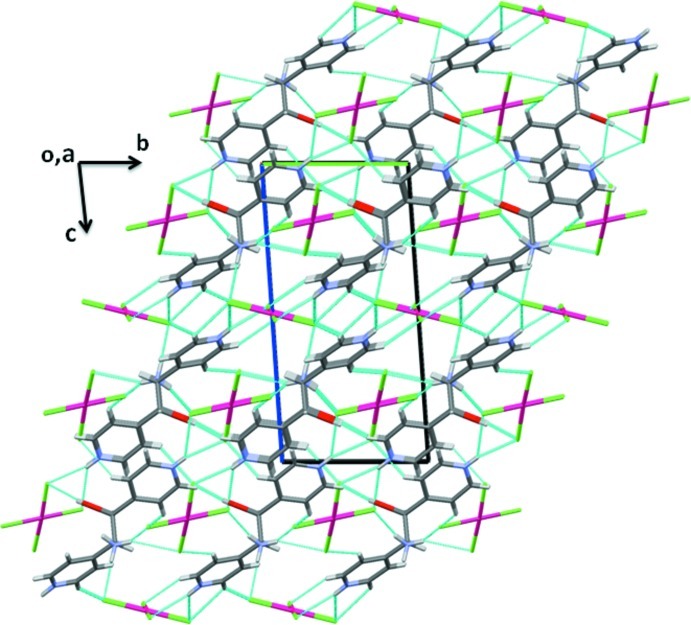
A view along the a axis of the crystal packing of the title compound, with the hydrogen bonds shown as dashed lines (see Table 1 for details).

## supplementary crystallographic information

### Comment

Hydrogen bond based organic–inorganic hybrid materials are receiving continuous interest because of their structural, magnetic, optical and electrical properties (Yao *et al.*, 2010; Sanchez *et al.*, 2011; Pardo *et al.*, 2011). An interesting approach for the preparation of such materials consists in the utilization of supramolecular synthons containing charge-assisted N^+^–H···Cl^-^ hydrogen bonds, through which organic cations and anionic metal complexes are linked to form crystalline organic–inorganic hybrid solids (Kumar *et al.*, 2006; Adams *et al.*, 2005,2006; Maris, 2008). As a further contribution we report herein on the crystal structure of the title compound.

The asymmetric unit of the title compound consists of one threefold charged organic cation in a general position and two independent [PdCl_4_]^2-^ dianions, one of which is located on a crystallographic inversion center (Fig. 1). In the cation, the pyridinium rings attached to the central 2-ammoniumethanol fragment show an *anti* conformation, as indicated by the C8—C1—C2—C3 torsion angle of -178.1 (4)°, and form a dihedral angle of 25.7 (2)°. The Pd atoms have square-planar coordination environments with Pd—Cl distances ranging from 2.2760 (10) to 2.3056 (11) Å.

In the crystal, the cations and anions are linked by N^+^—H···Cl^-^ and O—H···Cl^-^ hydrogen bonds (Table 1 and Fig. 2). There are π—π interactions present involving inversion related pyridinium rings [Cg···Cg^i^ distance = 3.788 (3) Å; Cg centroid of the N3,C3-C7 ring; symmetry code (i) -x, -y+2, -z]. There are also a number of C-H···O and C-H···Cl interactions present, consolidating the formation of a three-dimensional structure (Table 1 and Fig. 2).

### Experimental

The organic entities in the title compound are the product of partial hydrolysis starting from 2,4,5-tris(pyridin-4-yl)-4,5-dihydro-1,3-oxazole, which was synthesized according to a previously reported procedure (Campos-Gaxiola *et al.*, 2012). For the preparation of the palladium complex, a solution of 2,4,5-tris(pyridin-4-yl)-4,5-dihydro-1,3-oxazole (0.05 g, 0.16 mmol) in methanol and concentrated HCl (37%, 3 ml) was added dropwise to a stirring solution of potassium tetrachloridopalladate (0.05 g, 0.16 mmol) in water (5 ml). The resulting yellow solution was stirred for 30 min at 333 K, whereupon the solution was left to evaporate slowly at room temperature. After two weeks, red crystals were isolated [Yield: 30%]. Spectroscopic and TGA data for the title compound are available in the archived CIF.

### Refinement

C bound H atoms were positioned geometrically and constrained using the riding-model approximation [aryl C—H = 0.93 Å and *U*_iso_(H) = 1.2*U*_eq_(C)]. The N—H and O—H hydrogen atoms were located in difference Fourier maps and were refined with distance restraints: N-H = 0.86 (1) for NH_3_^+^ H atoms and 0.84 (1) Å for O—H and pyN-H^+^ H atoms, with *U*_iso_(H) = 1.2*U*_eq_(O,N).

### Crystal data

**Table:**

(C_12_H_16_N_3_O)_2_[PdCl_4_]_3_	*Z* = 1
*M**_r_* = 1181.16	*F*(000) = 576
Triclinic, *P*1	*D*_x_ = 2.166 Mg m^−^^3^
Hall symbol: -P 1	Mo *K*α radiation, λ = 0.71073 Å
*a* = 7.6970 (7) Å	Cell parameters from 3544 reflections
*b* = 7.7339 (7) Å	θ = 2.7–28.2°
*c* = 15.7254 (13) Å	µ = 2.40 mm^−^^1^
α = 84.541 (2)°	*T* = 100 K
β = 81.314 (2)°	Rectangular prism, red
γ = 78.717 (1)°	0.29 × 0.22 × 0.17 mm
*V* = 905.40 (14) Å^3^	

### Data collection

**Table:**

Bruker SMART CCD area-detector diffractometer	3143 independent reflections
Radiation source: fine-focus sealed tube	2927 reflections with *I* > 2σ(*I*)
Graphite monochromator	*R*_int_ = 0.019
phi and ω scans	θ_max_ = 25.0°, θ_min_ = 1.3°
Absorption correction: multi-scan (*SADABS*; Sheldrick, 1996)	*h* = −9→7
*T*_min_ = 0.543, *T*_max_ = 0.686	*k* = −9→9
5043 measured reflections	*l* = −16→18

### Refinement

**Table:**

Refinement on *F*^2^	Primary atom site location: structure-invariant direct methods
Least-squares matrix: full	Secondary atom site location: difference Fourier map
*R*[*F*^2^ > 2σ(*F*^2^)] = 0.032	Hydrogen site location: inferred from neighbouring sites
*wR*(*F*^2^) = 0.077	H atoms treated by a mixture of independent and constrained refinement
*S* = 1.07	*w* = 1/[σ^2^(*F*_o_^2^) + (0.0322*P*)^2^ + 2.1607*P*] where *P* = (*F*_o_^2^ + 2*F*_c_^2^)/3
3143 reflections	(Δ/σ)_max_ < 0.001
232 parameters	Δρ_max_ = 1.23 e Å^−^^3^
6 restraints	Δρ_min_ = −0.48 e Å^−^^3^

### Special details

**Table:**

Experimental. Spectroscopic and TGA data for the title compound: IR(KBr, cm^-1^): 3440, 3211, 3064, 2937, 2888, 1669, 1630, 1509, 1421, 1358, 1294, 999, 832. TGA: Calcd. for HCl: 3.08. Found: 3.26% (303–448 K); Calcd. for PdCl_2_: 15.49. Found: 15.85% (448–523 K).
Geometry. Bond distances, angles etc. have been calculated using the rounded fractional coordinates. All su's are estimated from the variances of the (full) variance-covariance matrix. The cell esds are taken into account in the estimation of distances, angles and torsion angles
Refinement. Refinement of *F*^2^ against ALL reflections. The weighted *R*-factor *wR* and goodness of fit *S* are based on *F*^2^, conventional *R*-factors *R* are based on *F*, with *F* set to zero for negative *F*^2^. The threshold expression of *F*^2^ > σ(*F*^2^) is used only for calculating *R*-factors(gt) *etc*. and is not relevant to the choice of reflections for refinement. *R*-factors based on *F*^2^ are statistically about twice as large as those based on *F*, and *R*- factors based on ALL data will be even larger.

### Fractional atomic coordinates and isotropic or equivalent isotropic displacement parameters (Å^2^)

**Table:**

	*x*	*y*	*z*	*U*_iso_*/*U*_eq_	
O1	−0.0806 (4)	0.6561 (4)	0.14345 (19)	0.0254 (10)	
N1	−0.3332 (5)	0.8197 (5)	0.2757 (2)	0.0243 (12)	
N2	0.1298 (5)	0.3847 (5)	0.4127 (2)	0.0229 (11)	
N3	−0.2750 (5)	1.2478 (5)	0.0153 (2)	0.0209 (11)	
C1	−0.1380 (6)	0.8184 (6)	0.2689 (3)	0.0240 (12)	
C2	−0.0541 (6)	0.8136 (6)	0.1750 (3)	0.0255 (12)	
C3	−0.1379 (6)	0.9732 (6)	0.1205 (3)	0.0221 (12)	
C4	−0.2352 (6)	0.9472 (6)	0.0571 (3)	0.0269 (14)	
C5	−0.3016 (6)	1.0887 (6)	0.0040 (3)	0.0250 (12)	
C6	−0.1864 (6)	1.2805 (6)	0.0775 (3)	0.0239 (12)	
C7	−0.1160 (6)	1.1430 (6)	0.1307 (3)	0.0219 (12)	
C8	−0.0458 (6)	0.6609 (6)	0.3204 (3)	0.0229 (12)	
C9	−0.1047 (6)	0.5003 (6)	0.3334 (3)	0.0259 (12)	
C10	−0.0142 (6)	0.3628 (6)	0.3807 (3)	0.0233 (12)	
C11	0.1937 (6)	0.5346 (6)	0.4001 (3)	0.0226 (12)	
C12	0.1067 (6)	0.6767 (6)	0.3539 (3)	0.0234 (12)	
Pd1	0.50000	1.00000	0.50000	0.0165 (1)	
Cl1	0.53138 (14)	0.71363 (13)	0.46422 (7)	0.0220 (3)	
Cl2	0.19678 (14)	1.02190 (13)	0.52122 (7)	0.0250 (3)	
Pd2	0.39126 (4)	0.32273 (4)	0.19078 (2)	0.0158 (1)	
Cl3	0.56147 (14)	0.25575 (14)	0.30002 (7)	0.0247 (3)	
Cl4	0.30607 (16)	0.05224 (14)	0.21810 (8)	0.0300 (3)	
Cl5	0.23446 (15)	0.37787 (13)	0.07476 (7)	0.0241 (3)	
Cl6	0.46316 (15)	0.60016 (13)	0.16265 (7)	0.0261 (3)	
H1	−0.12040	0.92890	0.29190	0.0290*	
H1'	0.014 (4)	0.584 (5)	0.130 (3)	0.0380*	
H1A	−0.391 (6)	0.924 (3)	0.265 (3)	0.0360*	
H1B	−0.361 (7)	0.742 (5)	0.247 (3)	0.0360*	
H1C	−0.374 (7)	0.788 (7)	0.3275 (12)	0.0360*	
H2	0.07710	0.81200	0.17130	0.0300*	
H2'	0.175 (6)	0.299 (4)	0.444 (3)	0.0350*	
H3'	−0.309 (6)	1.338 (4)	−0.016 (3)	0.0310*	
H4	−0.25600	0.83250	0.05030	0.0320*	
H5	−0.36640	1.07190	−0.04050	0.0300*	
H6	−0.17290	1.39760	0.08450	0.0290*	
H7	−0.05210	1.16390	0.17470	0.0260*	
H9	−0.20700	0.48570	0.30960	0.0310*	
H10	−0.05410	0.25300	0.39050	0.0280*	
H11	0.29880	0.54330	0.42300	0.0280*	
H12	0.15060	0.78450	0.34500	0.0280*	

### Atomic displacement parameters (Å^2^)

**Table:**

	*U*^11^	*U*^22^	*U*^33^	*U*^12^	*U*^13^	*U*^23^
O1	0.0310 (18)	0.0191 (15)	0.0257 (17)	−0.0019 (13)	−0.0048 (14)	−0.0037 (13)
N1	0.023 (2)	0.025 (2)	0.022 (2)	0.0003 (16)	−0.0014 (16)	0.0011 (16)
N2	0.022 (2)	0.0226 (19)	0.0203 (19)	0.0029 (15)	−0.0056 (15)	0.0074 (15)
N3	0.0170 (18)	0.0227 (19)	0.0197 (19)	−0.0009 (15)	−0.0016 (15)	0.0078 (15)
C1	0.022 (2)	0.026 (2)	0.025 (2)	−0.0060 (18)	−0.0078 (18)	0.0033 (19)
C2	0.022 (2)	0.026 (2)	0.028 (2)	−0.0038 (18)	−0.0023 (19)	−0.0030 (19)
C3	0.022 (2)	0.025 (2)	0.019 (2)	−0.0064 (18)	−0.0026 (18)	0.0042 (18)
C4	0.036 (3)	0.021 (2)	0.025 (2)	−0.009 (2)	−0.005 (2)	0.0004 (18)
C5	0.026 (2)	0.032 (2)	0.020 (2)	−0.011 (2)	−0.0088 (19)	0.0037 (19)
C6	0.029 (2)	0.022 (2)	0.021 (2)	−0.0080 (19)	−0.0011 (19)	0.0007 (18)
C7	0.022 (2)	0.029 (2)	0.018 (2)	−0.0110 (18)	−0.0060 (17)	0.0006 (18)
C8	0.024 (2)	0.023 (2)	0.022 (2)	−0.0039 (18)	−0.0070 (18)	0.0019 (18)
C9	0.029 (2)	0.023 (2)	0.028 (2)	−0.0077 (19)	−0.010 (2)	0.0029 (19)
C10	0.025 (2)	0.018 (2)	0.026 (2)	−0.0040 (18)	−0.0014 (19)	−0.0009 (18)
C11	0.018 (2)	0.030 (2)	0.019 (2)	−0.0020 (18)	−0.0055 (18)	0.0026 (18)
C12	0.022 (2)	0.026 (2)	0.023 (2)	−0.0082 (18)	−0.0049 (18)	0.0052 (18)
Pd1	0.0180 (2)	0.0144 (2)	0.0185 (2)	−0.0050 (2)	−0.0073 (2)	0.0038 (2)
Cl1	0.0290 (6)	0.0162 (5)	0.0224 (5)	−0.0075 (4)	−0.0071 (4)	0.0026 (4)
Cl2	0.0193 (5)	0.0206 (5)	0.0357 (6)	−0.0063 (4)	−0.0081 (4)	0.0079 (4)
Pd2	0.0177 (2)	0.0147 (2)	0.0151 (2)	−0.0023 (1)	−0.0044 (1)	0.0000 (1)
Cl3	0.0261 (6)	0.0283 (5)	0.0216 (5)	−0.0071 (4)	−0.0102 (4)	0.0034 (4)
Cl4	0.0375 (6)	0.0182 (5)	0.0389 (7)	−0.0095 (5)	−0.0204 (5)	0.0085 (5)
Cl5	0.0303 (6)	0.0203 (5)	0.0245 (5)	−0.0067 (4)	−0.0132 (4)	0.0031 (4)
Cl6	0.0338 (6)	0.0204 (5)	0.0286 (6)	−0.0114 (4)	−0.0137 (5)	0.0039 (4)

### Geometric parameters (Å, º)

**Table:**

Pd1—Cl2	2.2821 (11)	C1—C8	1.514 (7)
Pd1—Cl1^i^	2.2950 (10)	C2—C3	1.526 (7)
Pd1—Cl1	2.2950 (10)	C3—C4	1.385 (7)
Pd1—Cl2^i^	2.2821 (11)	C3—C7	1.383 (6)
Pd2—Cl4	2.2960 (12)	C4—C5	1.376 (7)
Pd2—Cl5	2.2985 (12)	C6—C7	1.366 (7)
Pd2—Cl3	2.2760 (12)	C8—C9	1.391 (7)
Pd2—Cl6	2.3056 (11)	C8—C12	1.388 (7)
O1—C2	1.419 (6)	C9—C10	1.373 (7)
O1—H1'	0.84 (4)	C11—C12	1.375 (7)
N1—C1	1.488 (6)	C1—H1	1.0000
N2—C10	1.330 (6)	C2—H2	1.0000
N2—C11	1.332 (6)	C4—H4	0.9500
N3—C5	1.319 (6)	C5—H5	0.9500
N3—C6	1.342 (6)	C6—H6	0.9500
N1—H1C	0.86 (2)	C7—H7	0.9500
N1—H1A	0.86 (3)	C9—H9	0.9500
N1—H1B	0.86 (4)	C10—H10	0.9500
N2—H2'	0.84 (4)	C11—H11	0.9500
N3—H3'	0.84 (4)	C12—H12	0.9500
C1—C2	1.521 (7)		
			
Cl1^i^—Pd1—Cl2^i^	89.74 (4)	C3—C4—C5	119.5 (4)
Cl1—Pd1—Cl2^i^	90.26 (4)	N3—C5—C4	119.6 (4)
Cl1—Pd1—Cl2	89.74 (4)	N3—C6—C7	119.1 (4)
Cl1—Pd1—Cl1^i^	180.00	C3—C7—C6	120.0 (4)
Cl1^i^—Pd1—Cl2	90.26 (4)	C1—C8—C9	122.8 (4)
Cl2—Pd1—Cl2^i^	180.00	C9—C8—C12	118.9 (4)
Cl4—Pd2—Cl5	89.59 (4)	C1—C8—C12	118.3 (4)
Cl3—Pd2—Cl6	92.45 (4)	C8—C9—C10	119.6 (4)
Cl3—Pd2—Cl4	89.51 (4)	N2—C10—C9	119.4 (4)
Cl4—Pd2—Cl6	177.33 (4)	N2—C11—C12	119.7 (4)
Cl5—Pd2—Cl6	88.58 (4)	C8—C12—C11	119.3 (4)
Cl3—Pd2—Cl5	176.06 (4)	C8—C1—H1	109.00
C2—O1—H1'	114 (3)	N1—C1—H1	109.00
C10—N2—C11	123.0 (4)	C2—C1—H1	109.00
C5—N3—C6	123.1 (4)	C3—C2—H2	109.00
C1—N1—H1C	110 (4)	C1—C2—H2	109.00
C1—N1—H1A	111 (3)	O1—C2—H2	109.00
H1A—N1—H1B	113 (4)	C3—C4—H4	120.00
C1—N1—H1B	115 (4)	C5—C4—H4	120.00
H1B—N1—H1C	102 (5)	N3—C5—H5	120.00
H1A—N1—H1C	106 (5)	C4—C5—H5	120.00
C10—N2—H2'	115 (3)	C7—C6—H6	120.00
C11—N2—H2'	122 (3)	N3—C6—H6	121.00
C6—N3—H3'	113 (3)	C3—C7—H7	120.00
C5—N3—H3'	124 (3)	C6—C7—H7	120.00
N1—C1—C8	111.3 (4)	C8—C9—H9	120.00
C2—C1—C8	109.4 (4)	C10—C9—H9	120.00
N1—C1—C2	110.0 (4)	N2—C10—H10	120.00
O1—C2—C3	109.6 (4)	C9—C10—H10	120.00
O1—C2—C1	107.9 (4)	N2—C11—H11	120.00
C1—C2—C3	111.6 (4)	C12—C11—H11	120.00
C2—C3—C7	122.2 (4)	C11—C12—H12	120.00
C4—C3—C7	118.7 (4)	C8—C12—H12	120.00
C2—C3—C4	119.1 (4)		
			
C11—N2—C10—C9	−1.0 (7)	C1—C2—C3—C4	−114.2 (5)
C10—N2—C11—C12	1.7 (7)	C1—C2—C3—C7	67.6 (6)
C6—N3—C5—C4	−0.7 (7)	C2—C3—C4—C5	−175.9 (4)
C5—N3—C6—C7	1.7 (7)	C7—C3—C4—C5	2.4 (7)
N1—C1—C2—O1	−61.0 (5)	C2—C3—C7—C6	176.8 (4)
N1—C1—C2—C3	59.5 (5)	C4—C3—C7—C6	−1.4 (7)
C8—C1—C2—O1	61.5 (5)	C3—C4—C5—N3	−1.4 (7)
C8—C1—C2—C3	−178.1 (4)	N3—C6—C7—C3	−0.6 (7)
N1—C1—C8—C9	30.7 (6)	C1—C8—C9—C10	179.9 (4)
N1—C1—C8—C12	−151.1 (4)	C12—C8—C9—C10	1.7 (7)
C2—C1—C8—C9	−91.0 (5)	C1—C8—C12—C11	−179.3 (4)
C2—C1—C8—C12	87.2 (5)	C9—C8—C12—C11	−1.0 (7)
O1—C2—C3—C4	5.3 (6)	C8—C9—C10—N2	−0.7 (7)
O1—C2—C3—C7	−172.9 (4)	N2—C11—C12—C8	−0.6 (7)

Symmetry code: (i) −*x*+1, −*y*+2, −*z*+1.

### Hydrogen-bond geometry (Å, º)

**Table:**

*D*—H···*A*	*D*—H	H···*A*	*D*···*A*	*D*—H···*A*
O1—H1′···Cl5	0.84 (4)	2.22 (4)	3.047 (3)	168 (4)
N1—H1*A*···Cl3^ii^	0.86 (3)	2.62 (2)	3.355 (4)	145 (4)
N1—H1*A*···Cl4^ii^	0.86 (3)	2.54 (4)	3.203 (4)	135 (4)
N1—H1*B*···Cl6^iii^	0.86 (4)	2.49 (5)	3.310 (4)	160 (4)
N1—H1*C*···Cl1^iii^	0.86 (2)	2.22 (2)	3.080 (3)	177 (6)
N2—H2′···Cl2^iv^	0.84 (4)	2.35 (4)	3.137 (4)	157 (4)
N3—H3′···Cl5^v^	0.84 (4)	2.44 (4)	3.150 (4)	143 (4)
N3—H3′···Cl6^v^	0.84 (4)	2.71 (5)	3.353 (4)	135 (3)
C4—H4···Cl5^vi^	0.95	2.64	3.406 (5)	139
C6—H6···O1^vii^	0.95	2.54	3.454 (6)	161
C9—H9···Cl3^iii^	0.95	2.78	3.599 (5)	145
C10—H10···Cl2^viii^	0.95	2.75	3.649 (5)	159
C11—H11···Cl1	0.95	2.61	3.486 (5)	154
C11—H11···Cl1^ix^	0.95	2.80	3.422 (5)	124

Symmetry codes: (ii) *x*−1, *y*+1, *z*; (iii) *x*−1, *y*, *z*; (iv) *x*, *y*−1, *z*; (v) −*x*, −*y*+2, −*z*; (vi) −*x*, −*y*+1, −*z*; (vii) *x*, *y*+1, *z*; (viii) −*x*, −*y*+1, −*z*+1; (ix) −*x*+1, −*y*+1, −*z*+1.
